# Clinical breast exam contribution to breast cancer diagnosis in *BRCA* mutation carriers vs. average to intermediate risk women

**DOI:** 10.1007/s10549-024-07345-3

**Published:** 2024-05-26

**Authors:** Tehillah S. Menes, Douglas Zippel, Miri Sklair-Levy, Eitan Friedman, Rinat Bernstein-Molho, Renata Faermann, Dana Madorsky Feldman

**Affiliations:** 1 Department of Surgery, Sheba Medical Center, Ramat Gan, Israel; 2 Meirav Center for Women’s Health and High-Risk Clinic, Sheba Medical Center, Ramat Gan, Israel; 3 Division of Diagnostic Imaging, Sheba Medical Center, Tel Hashomer, Ramat Gan, Israel; 4 Tel Aviv School of Medicine, Tel Aviv University, Tel Aviv-Yafo, Israel

**Keywords:** Breast cancer, Clinical breast exam, BRCA, Surveillance

## Abstract

**Purpose:**

The contribution of clinical breast exam (CBE) to breast cancer diagnosis in average risk women undergoing regular screening mammography is minimal. To evaluate the role of CBE in high-risk women, we compared BC diagnosis by CBE in *BRCA* mutation carriers undergoing regular BC surveillance to average to intermediate risk women undergoing regular breast cancer screening.

**Methods:**

A retrospective chart review of all consecutive screening visits of *BRCA* mutation carriers (January 2012–October 2022) and average to intermediate risk women (November 2016–December 2022) was completed. Women with histologically confirmed BC diagnosis were included. Additional CBE yield for BC diagnosis, defined as the percentage of all BC cases detected by CBE alone, was assessed in both groups.

**Results:**

Overall, 12,997 CBEs were performed in 1,328 *BRCA* mutation carriers in whom 134 BCs were diagnosed. In 7,949 average to intermediate risk women who underwent 15,518 CBEs, 87 BCs were diagnosed. CBE contributed to BC diagnosis in 3 (2%) *BRCA* mutation carriers and 3 (4%) non-carriers. In both groups, over 4,000 CBEs were needed in order to diagnose one cancer. In all 3 *BRCA* mutation carriers BC was palpated during the surveillance round that did not include MRI. In the average to intermediate risk group, 2 of 3 cancers diagnosed following CBE findings were in a different location from the palpable finding.

**Conclusions:**

The contribution of CBE to BC diagnosis is marginal for all women including *BRCA* mutation carriers. In *BRCA* mutation carriers, CBE appears redundant during the MRI surveillance round.

## Introduction

With improvements in breast imaging, the role of clinical breast exam (CBE) in screening average risk women is increasingly being questioned; with a recent report suggesting a very low yield in women undergoing regular mammography screening [1]. The American Cancer Society (ACS) does not recommend CBE for BC screening in average risk women at any age [2]. This recommendation is based on lack of level 1 evidence of any proven benefit for CBE either as a stand-alone tool or in conjunction with screening mammography [3]. The National Cancer Comprehensive Network [4] recommends a clinical encounter starting at age 25, which includes a CBE in asymptomatic individuals. Despite lack of proof of efficacy of CBE in average risk women, it may play a role in high-risk women, specifically *BRCA* mutation carriers. Inclusion of CBE in high-risk surveillance protocols varies worldwide, with some societies recommending annual or bi-annual CBE, while others do not [5, 6]. The ESMO recent recommendations [7] for surveillance of *BRCA* mutation carriers clearly state that “Clinical breast examination is of no value as a screening tool”, referencing a report by Hettipathirana et al [8]. In their report, 35 cancers were diagnosed in 414 *BRCA* mutation carriers between 2001 and 2019. Of these, two BCs were detected by CBE alone; however, this was prior to the introduction of MRI into the surveillance protocol in this center in 2009.

We sought to examine the role of CBE in early stage BC diagnosis in high-risk women undergoing regular BC surveillance. As the yield of CBE in women undergoing regular screening mammography is minimal [1], we compared BC detection by CBE alone in *BRCA* mutation carriers to women at average to intermediate risk undergoing regular breast cancer screening in the same medical center.

## Methods

This retrospective chart review was approved by the local ethics committee (SMC-21-8844), and informed consent was waived.

### Study population

All consecutive visits of women to the high-risk clinic (January 2012 to October 2022) or to the health screening clinic (November 2016 to December 2022) were identified using MDClone (MDClone LTD, Beer Sheva, Israel), a query tool providing a wide range of patient data.

The High-risk clinic at Sheba Medical Center, Tel Hashomer, provides BC screening to high-risk women, predominantly *BRCA* pathogenic mutation carriers. Only women with a known pathogenic mutation in *BRCA1/2* gene were included in the study group. *BRCA* mutation carriers with a previous history of BC were included in the cohort, as once completing successful treatment high-risk surveillance is continued as per *BRCA* surveillance protocol.

The control group consisted of all consecutive women visiting the health screening center at Sheba Medical Center Tel Hashomer. The program is voluntary and provides general health screening services. Most participants receive the service as an employer-provided benefit. Women examined in the health screening clinic were excluded if they had a previous diagnosis of BC (as these women are considered to be at risk for both local recurrence and a new primary and recommendations for surveillance of these women include CBE and more frequent imaging).

Based on their family history the control group included women at average and intermediate risk for breast cancer. Women who reported any family history of breast cancer without a known pathogenic mutation were considered to be at intermediate risk. However, women with a strong family history suggestive of hereditary cancer syndrome were excluded due to their high-risk.

### Surveillance and screening protocols

The surveillance protocol for *BRCA* pathogenic mutation carriers includes CBE every 6 months starting at the age of 25; and annual breast MRI starting at the age of 25 years, alternating with annual ultrasound (US) or mammography (starting at age 30). Pregnant and breast feeding women undergo CBE and US every 3 months. Women that are post risk-reducing bilateral mastectomy continue to undergo CBE every 6 months, and a baseline MRI. After assessment of residual breast tissue individual recommendations are given.

The breast screening protocol at the screening center includes a yearly CBE by a surgeon and imaging according to personal risk. This usually includes a yearly screening mammogram from age 40 and breast ultrasound (US) or contrast enhanced spectral mammography (CESM) based on mammographic density. There is no upper age limit for CBE or screening mammography. Some of the women undergo regular breast screening by their HMO and complete only part of the breast health exam during their visit at the screening center. In women with findings on CBE, further evaluation is recommended as indicated by the surgeon and/or the breast radiologist.

Women presenting with a symptomatic cancer, i.e., complaining of symptoms that were related to the cancer, were considered to have an interval cancer.

### Breast imaging

Mammography was performed with a digital mammography system (Senographe Essential and Senographe Pristina; GE Healthcare, New York, NY, USA). Standard craniocaudal and mediolateral oblique projections of each breast were acquired. CESM studies were performed using a digital mammography system (Senographe Essential, GE Healthcare; Chalfont St-Giles, UK). Whole breast and bilateral axillae US examinations were performed by certified fellowship-trained breast radiologists, using Siemens Acuson S2000 ultrasound system with a linear transducer 18-6MHz, (Siemens Medical Solutions USA, Inc., Mountain View, CA, USA). MRI was performed on 1.5-T MRI (Signa Excite HDX, GE Healthcare) using a dedicated double breast coil.

The imaging was considered abnormal if the Breast Imaging-Reporting and Data System (BIRADS) classification was 0, 3, 4, or 5, or if the text included a recommendation for further work-up.

### Data collection

Using ICD-9 codes, the charts of women with a diagnosis of BC were identified. Further data were manually extracted from the electronic medical records (Chameleon, version 5.12.2.43395, Elad Health, Israel).

Women that were diagnosed with breast cancer after a visit to one of the clinics were included in the final cohort.

Data retrieved from the charts included demographics, family history of cancer, time from previous imaging, clinical and imaging findings, tumor features and treatment.

Based on the chart review, the mode of diagnosis was determined. If a palpable finding led to further work-up and a subsequent diagnosis of BC, this was considered to be BC diagnosed by CBE. If the finding on CBE correlated with a finding on standard imaging, this was not considered BC diagnosed by CBE. As the tests are performed in parallel with some women first undergoing a CBE while others undergo imaging first, charts were manually reviewed in order to determine if a finding on CBE led to the diagnosis of BC (i.e., second look US, etc.).

### Analysis

Descriptive statistics of the study population were summarized. The two groups were compared using chi-square test for categorical data, and the Student’s *t*-test for continuous variables. All tests were 2-sided and significance was set at 0.05. The additional cancer yield of CBE (defined as the percentage of all cancer cases detected by CBE only) and number needed to screen by CBE in order to diagnose one cancer were calculated. These proportions were calculated separately for the two groups. Rate precision was determined with 95% confidence intervals (CI), which were derived by using the Wilson score interval for binomial parameters with continuity correction.

## Results

**Fig. 1:**
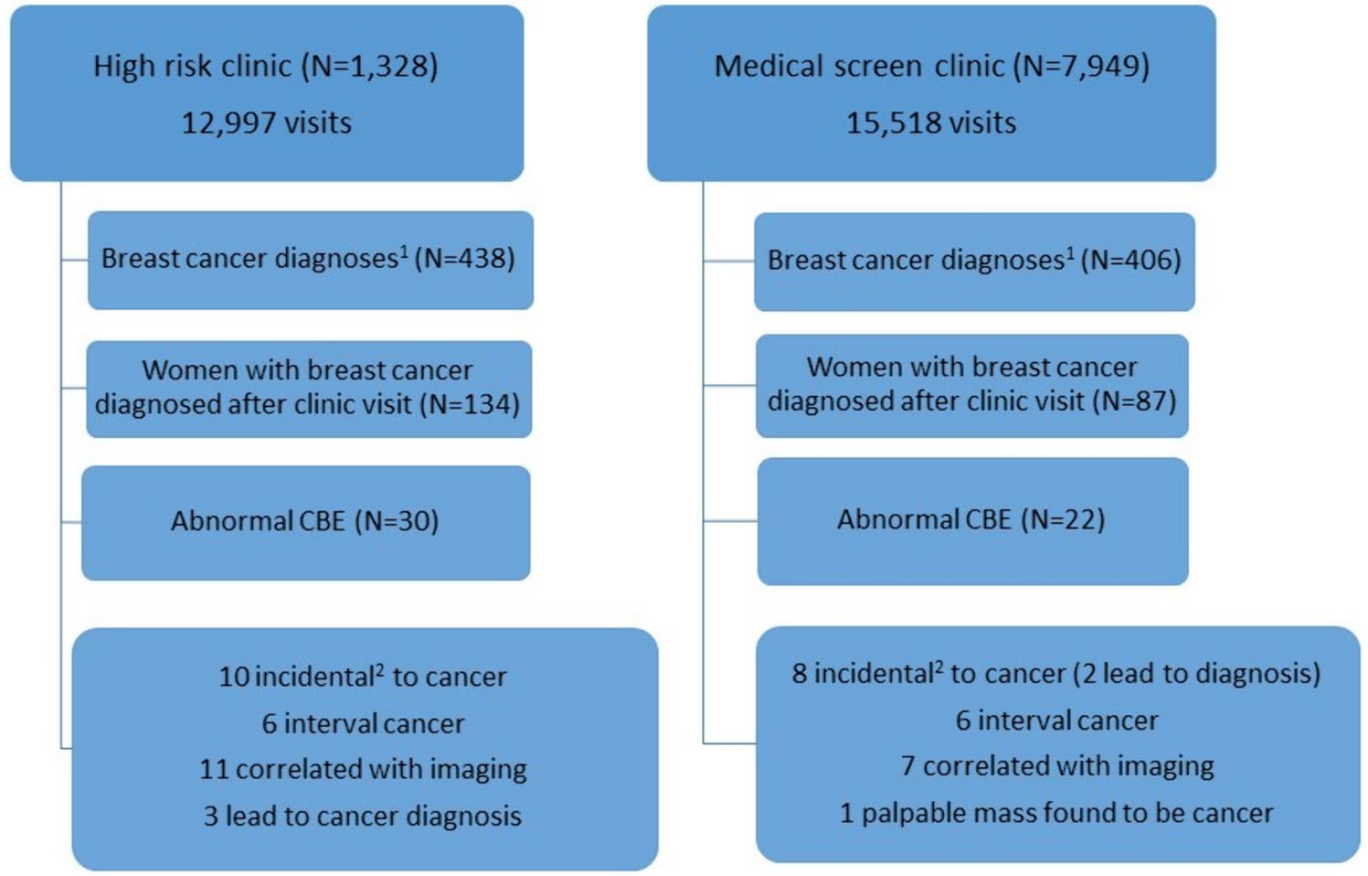
Flow chart of study cohorts. ^1^ Including breast cancers that were diagnosed prior to the study and therefore excluded. ^2^ Incidental- the clinical abnormality was not at the location of the cancer

During the study period, 1,328 *BRCA* mutation carriers (*BRCA*1 – 773; *BRCA*2- 550; *BRCA*1 + *BRCA*2 – 5) had 12,997 documented CBEs. After excluding BCs that were diagnosed prior to the first high-risk clinic visit, the cohort included 134 BC cases in the *BRCA* mutation carrier group (Fig. 1, Table 1). In 39 (29%) of these women, there was at least one previous diagnosis of breast cancer. Five were post bilateral mastectomy. Five had a history of ovarian cancer. Seventy (52%) underwent bilateral salpingo-oopherectomy prior to the current diagnosis. Mean time from previous imaging was 0.56 years (range 0.03–1.47 years). Findings on CBE are summarized in Fig. 1.

Six (4.5%) women presented with interval cancers, median age was 36 years (range 28–67). Median time from previous imaging was 3 months (range 0.11–0.66). Time from previous MRI ranged between 7.5 months and 3 years; one woman had never had a previous MRI. Average tumor size was 18 mm (range 10–46mm). Four were node negative, one was node positive, and for one this data was missing. Two were triple negative, two were luminal, one was HER2neu positive and data were missing for the last.

Three cancers (2%; 95% CI 0.7–6) were diagnosed secondary to CBE findings, none of these women had an MRI during this round (Table 2). In the screening rounds not including MRI three of the BCs were diagnosed by CBE (7.7%; 95% CI 2.6–20.3).

In the Health screening center 7,949 average to intermediate risk women had 15,518 CBEs done during the study period. The final cohort included 87 women diagnosed with BC after a visit to the clinic with a documented CBE within 6 months of the diagnosis (Fig. 1).

Most of these women (54; 62%) had no known family history of BC (Table 1). Four women with no known family history were subsequently identified as *BRCA* mutation carriers.

**Table 1:**
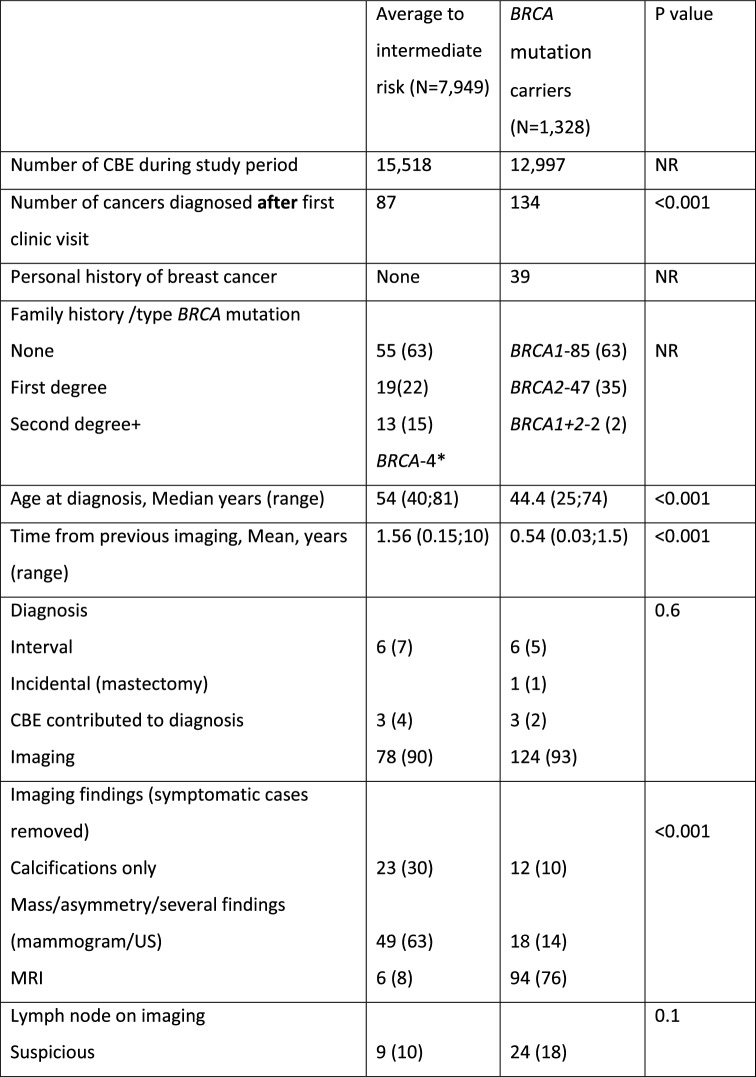

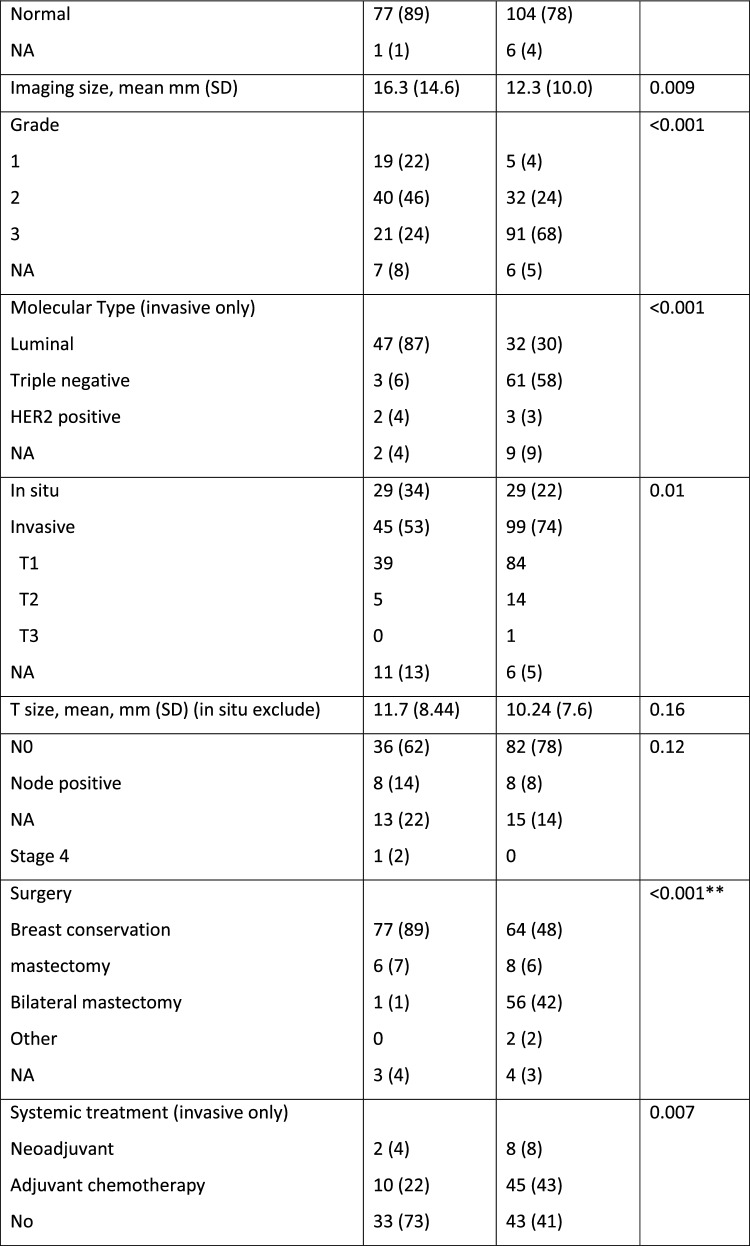

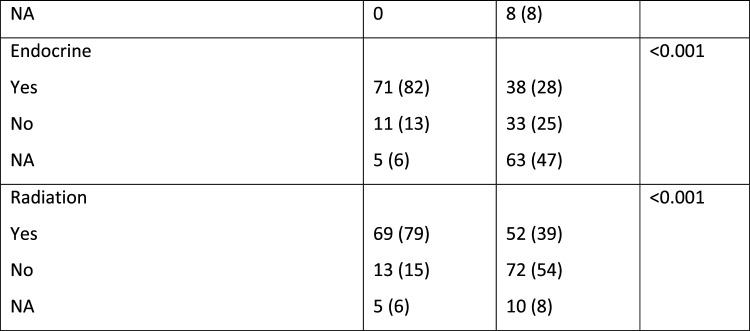
Comparison of average to intermediate risk group with *BRCA* mutation carrier group

*Post diagnosis genetic testing

**For BCT vs. all mastectomy

The imaging, pathology and treatment characteristics are summarized in Table 1.

The median age of women presenting with an interval cancer was 64.3 years (range 41–81). Median time from previous imaging was 0.8 years (range 0.73–10).

There were 3 (4%; 95% CI 1–9) cancers that were diagnosed secondary to an abnormal CBE (Table 2). One was found on MRI; the other 2 on US. In two of these cases, the palpable finding was in a different location from the cancer diagnosed. Nonetheless, the CBE initiated the work-up.

**Table 2:** Cancers diagnosed secondary to abnormal CBE

	Age	Previous imaging	Time from previous imaging (months)	Location of cancer	Pathology	type	T size (mm)	Node status
*BRCA1*	33	MRI	6	Palpable	IDC	TN	25	0
*BRCA2*	40	MRI	6	Palpable	IDC	TN	17	0
*BRCA2*	33	US	3.5	Palpable	IDC	Luminal	7	0
Average risk	60	Mammogram + US	6	Palpable	IDC	Luminal	19	Mic
Average risk	70	Mammogram + US	4	Incidental (MRI)	IDC	Luminal	8	0
Average risk	68	Mammogram + US	10	Incidental (US)	IDC	Luminal	6	0

As expected, when compared to the average to intermediate risk group, *BRCA* mutation carriers were diagnosed with BC at an earlier age; mainly by MRI; and more often with invasive, high grade triple negative cancers. They underwent more often mastectomies and received systemic chemotherapy (Table 1).

In both groups, diagnosis of cancer by CBE only was a rare event; over 4,000 exams were needed in order to diagnose one BC.

## Discussion

In order to assess the yield of CBE in high-risk women we compared the additional cancer yield of CBE in BRCA carriers to average to intermediate risk women undergoing regular screening mammography. In the current study, the additional yield of CBE to BC diagnosis in both *BRCA* mutation carriers and in average to intermediate risk population was marginal at best. Notably, the number of CBE needed in order to find one breast cancer was over 4,000 in *BRCA* mutation carriers. These results are in line with previous studies in which the additional yield of CBE to BC detection ranged from 0 to 6% (Table 3). The incorporation of breast MRI in the surveillance protocol of *BRCA* mutation carriers resulted in a decrease in the proportion of women presenting with interval cancers from 35–50% [9] to 0–19% [10–13, 15, 16] (Table 3).

**Table 3:** Studies reporting cancers detected in *BRCA* mutation carriers by CBE

Study (number of *BRCA* mutation carriers)	Study years	cancers detected	Detected by CBE (%)	Mammogram ± US^a^	MRI^a^	interval	Incidental at surgery	Node positive (%)
Warner [10] 2004 (*N* = 236)	1997–2003	22	0	15	17	1	1	2 (9)
Kriege [11] 2004 (358)	1999–2003	23	NA	NA	NA	3	1	7 (30)
Trop [12] 2010 (*N* = 143)	2003–2007	11	0	7	9	0	0	1 (9)
Rijnsburger [13] 2010 (*N* = 594)	1999–2006	53^c^	1 (2)	14	24	10	6	14 (26)
Maurice [14] 2011 (*N* = 251)	1987–2008	45	2(4)	28	ND	11	Excluded	13 (29)
Fakkert [15] 2011 (*N* = 139)	1995–2009	14	0	5	6	2	0	6 (43)
Mihalco [16] 2020 (*N* = 88)	2016	3	0	ND	3	0	0	NA
Hettipathirana8 2021 (*N* = 414)	2001–2019	35	2^e^(6)	6	21	7	1	4 (15)
Current (*N* = 1328)	2012–2022	134	3 (2)	30	95	6	1	8 (6)

^a^diagnoses on different imaging modalities may overlap

^b^including PTEN, TP53

^c^imaging data available only on 37

^d^post risk reducing salpingo-oopherectomy

^e^MRI and US not performed in all cases

In 2014, Roeke et al. [17] systematically reviewed the additional cancer yield of CBE in women at increased risk of BC, and reported that it ranged between 0 and 4% in 7 prospective studies. The recent ESMO guidelines removed the previous recommendation for CBE in surveillance of *BRCA* mutation carriers, while recommending in *BRCA1* mutation carriers imaging every 6 months preferably by MRI [7]. The National Institute of Health and Care Excellence (NICE) continues to recommend breast awareness and annual MRI [18], while NCCN continues to recommend CBE from age 25 and annual MRI [19].

Based on the results of the current study, and given the well- established superiority of MRI over other breast imaging modalities [20], combined with the fact that all 3 BC cases in *BRCA* mutation carriers identified by CBE were in women not undergoing MRI at the same screening round, it appears safe to forgo the CBE at the time surveillance MRI is performed.

This study has several limitations. Data on visits to the high-risk clinic and the health screening center were based on computer queries and therefore errors in coding may have resulted in inaccurate estimation of the total number of women and of the total number of visits to these clinics. Although charts of women subsequently diagnosed with breast cancer were manually reviewed, the total number of women and of visits was based on retrieval of visit codes. This may impact the accuracy of the estimation of the number needed to screen in order to detect one BC. As the estimates of the additional cancer yield of CBE are based on women diagnosed with breast cancer, these estimates are minimally affected by coding errors. The *BRCA* cohort included women after a previous diagnosis of BC (as these women are recommended to continue the same surveillance scheme), whereas we excluded women with a history of BC in the average to intermediate risk group. Women that underwent bilateral mastectomy were included in the *BRCA* cohort despite controversy regarding their continued need for increased surveillance. With the increasing prevalence of risk-reducing mastectomy, CBE may play a bigger role in the surveillance of these women, however our numbers are too small to reach meaningful conclusions. The surgeons performing the CBEs were a heterogenous group, with varying experience and abilities. CBE is a skill which is almost impossible to quantify and analyze objectively. The population analyzed herein is from a single medical center in Israel and may not reflect the reality in other medical centers in the country. The compliance of average risk women to BC screening is below 80% in Israel and this may have impacted the results as well.

In summary, based on a large population of *BRCA* mutation carriers, it appears that CBE has a marginal contribution to the diagnosis of BC, specifically during the screening round that does not include MRI. It seems safe not to perform CBE during the screening visit that includes an MRI. In average to intermediate risk women undergoing regular BC screening the yield of CBE is very low, and may not be justified at all.

## Data Availability

The datasets generated and/or analyzed during the current study are not publicly available due privacy concerns. Upon request the analyses generated from the data are available from the corresponding author.
